# Synthesis of dual-modified Fe-doped and carbon-coated Li_4_Ti_5_O_12_ anode based on industrial H_2_TiO_3_ for Li-ion batteries

**DOI:** 10.1038/s41598-023-41830-x

**Published:** 2023-09-13

**Authors:** Xinyu Jiang, Guangqiang Ma, Qinmei Zhu, Hongwei Ge, Qiyuan Chen, Beilei Yan, Lin Deng, Congxue Tian, Chuanbao Wu

**Affiliations:** 1 College of Biological and Chemical Engineering, Panzhihua University, Panzhihua, 617000 People’s Republic of China; 2 Jiangxi Provincial Key Laboratory of Functional Molecular Materials Chemistry, School of Chemistry and Chemical Engineering, Jiangxi University of Science and Technology, Ganzhou, 341000 People’s Republic of China

**Keywords:** Energy science and technology, Materials science, Chemistry, Electrochemistry, Energy, Materials chemistry

## Abstract

Spinel Li_4_Ti_5_O_12_ (LTO) is a promising candidate for lithium-ion battery anodes because of its exceptional stability and safety. However, its extensive application is limited by a high comprehensive cost, poor electronic conductivity, and other inherent defects. This work presents a novel synthesis procedure to synthesize carbon-coated Fe-doped LTO composites through carbon reduction, in the presence of Fe-containing industrial H_2_TiO_3_ as the titanium source, and glucose as the carbon source. The presence of the Fe-dopant is confirmed through XRD, with Rietveld refinement and EDS experiments. Results show that Fe^2+^ replaces a portion of Ti^4+^ after doping, leading to an increase in the LTO cell parameters and the corresponding cell volume. FLTO/C, presents a capacity of 153.79 mAh g^−1^ at 10 C, and the capacity decay per cycle is only 0.0074% after 1000 cycles at 5 C. Moreover, EIS experiments indicate that the incorporation of Fe and carbon lowers the charge transfer resistance and improves the diffusion and migration of Li^+^. Notably, since this preparation process requires no additional Fe source as a raw material, it is simple, cost-effective, and suitable for large-scale production and further application.

## Introduction

Over the past decade, lithium-ion batteries (LIBs) have been extensively applied across various fields, due to their advantageous high energy density, long lifespan, rapid charge–discharge, and other advantages^1–3^. However, commercial graphite anode materials are limited by short lifespan and poor safety performance, thereby limiting further development of LIBs^4–6^. Thus, developing new anode materials to replace the traditional graphite anode is essential for overcoming the existing bottlenecks regarding the circulation and safety of LIBs.

Spinel LTO-based anodes are considered the most promising alternative to graphite. LTO is environmentally friendly and has good stability, thus, it is, commonly referred to as a “zero-strain material,” and shows an extended lifespan during the lithiation/de-lithiation process. Additionally, the LTO anode provides a stable voltage platform of ~ 1.55 V vs Li^+^/Li during the Li-ion lithiation/de-lithiation process, thereby preventing the formation of lithium dendrites^5, 7, 8^. However, while LTO shows the highest safety and durability among anode candidates, it has a low specific capacity (175 mAh g^−1^), poor electrical conductivity (10^−13^ S cm^−1^), and high production costs^9, 10^. Various strategies have been suggested to overcome the limitations of LTO, such as carbon coating^11, 12^, morphology control^13, 14^, ion doping^15–18^ and finding cheaper raw materials to prepare LTO^19^.

Metatitanic acid (H_2_TiO_3_) is an intermediate product in the industrial preparation of titanium dioxide, using the sulfuric acid method. The impurity content of Fe in conventional H_2_TiO_3_ used in industries ranges from 200 to 1500 ppm^20–22^, as some residual Fe ions can be adsorbed into the H_2_TiO_3_ structure. Based on previous studies on the preparation of Li_4_Ti_5_O_12_ by industrial H_2_TiO_3_^23^, this work, industrial H_2_TiO_3_ is used as the titanium source, and directly used to prepare Fe-doped and carbon-coated LTO composite through a high-temperature solid-phase method. Compared to conventional Fe-doped preparation methods, our technique does not require additional Fe-containing compounds and has low production costs, with no by-products. The dual-modified LTO exhibited substantially enhanced electrochemical performance, with a specific capacity reaching 159.50 mAh g^−1^ at 5 C and 153.79 mAh g^−1^ at 10 C, and a capacity retention rate of 92.56% after 1000 charge–discharge cycles at 5 C.

## Experimental section

### Material synthesis

**Figure 1:**
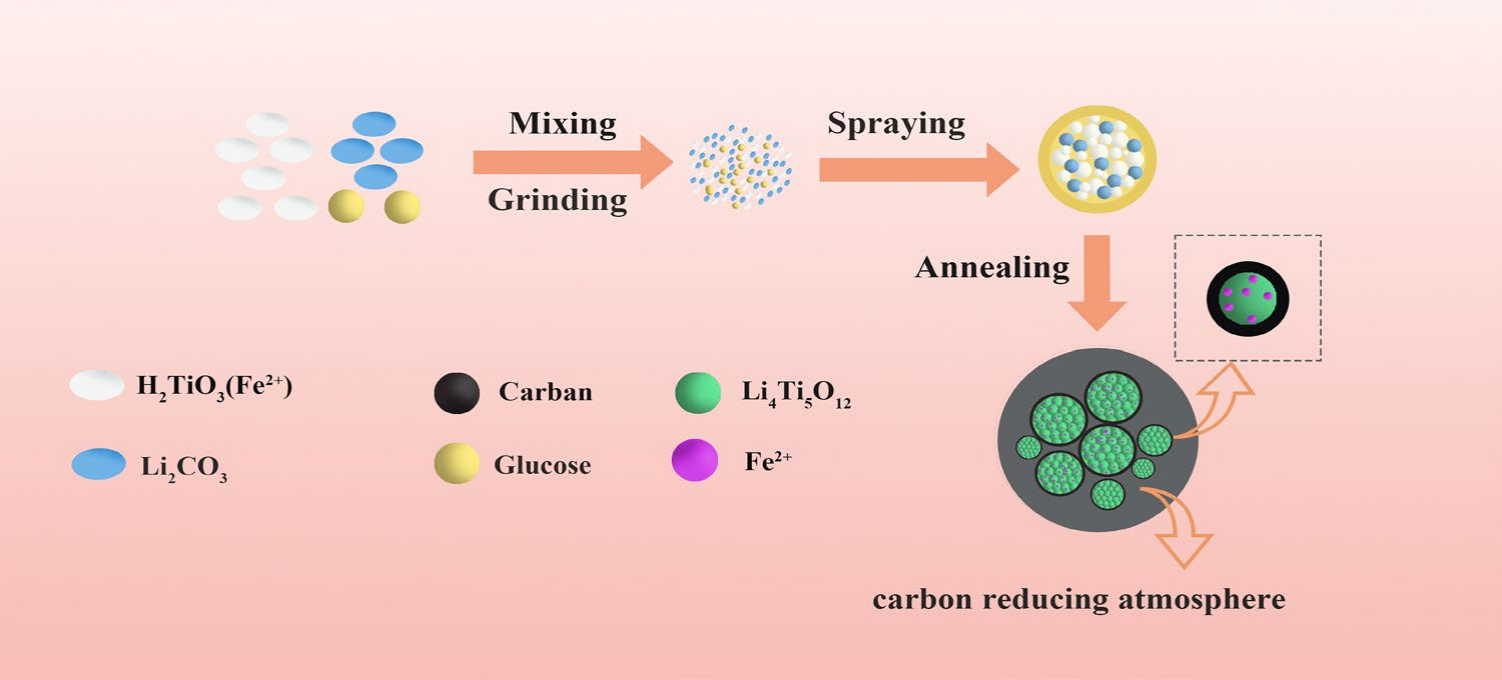
Schematic diagram for the fabrication process of the FLTO/C composites.

A schematic representation of the fabrication process of the FLTO/C composites is illustrated in Fig. 1. To obtain the composite, first, lithium carbonate (Li_2_CO_3_) was mixed with the industrial H_2_TiO_3_ (with 0.13% Fe content) at a certain lithium to titanium molar ratio of 0.93. Then, 15% of the total mass of glucose was weighed and utilized as the carbon source. The mixture was placed into a ball milling tank and ground for 2 h. After ball milling, the mixture was diluted with into a slurry (20%-ratio) with-deionized water and then spray dried with a spray drying inlet air temperature of 200 °C and a peristaltic pump speed of 24 rpm. Finally, the spray-dried precursor was placed in a 10 mL crucible, and then covered in a 120 mL crucible filled with rice husk charcoal. In the middle, the in-situ Fe-doped and carbon-coated Li_4_Ti_5_O_12_ (FLTO/C) samples were prepared by placing the buried precursor in a muffle furnace and calcination at 500 °C for 1 h and then at 800 °C for 8 h. As a control, the original Li_4_Ti_5_O_12_ (PLTO) was prepared with the same procedure, without added glucose and the titanium source used was analytically pure H_2_TiO_3_. Another control, titanium source using analytically pure industrial H_2_TiO_3_, and carbon-coated Li_4_Ti_5_O_12_ (PLTO/C) was synthesized based on the same process.

### Material characterization

A Bruker DX-2700 advanced diffractometer was employed to study the crystal structure and phase composition of the Li_4_Ti_5_O_12_ samples through XRD analysis. The carbon content of the Li_4_Ti_5_O_12_ samples was analyzed using the thermogravimetric (TG-DSC). The morphology and elemental distribution of the Li_4_Ti_5_O_12_ samples were characterized using of a scanning electron microscope (SEM, Hitachi SU8220) equipped with an energy dispersive spectrometer (EDS). The lattice spacing of the samples was observed using a transmission electron microscope (TEM, Talos F200S). To determine the chemical bonds present at the surface of the electrode materials, X-ray photoelectron spectroscopy (XPS), utilizing the ESCALAB Xi+, was employed.

### Electrochemical characterization

The active substance, acetylene black, and polyvinylidene fluoride were mixed at a mass ratio of 8:1:1 and dissolved in *N*-methyl-2-pyrrolidone (NMP). The mixture was stirred for 30 min to ensure uniform mixing. Subsequently, the mixed slurry was evenly coated on the copper foil of double bread carbon using a 90 μm scraper and then transferred to a vacuum drying oven at 80 °C for 24 h. The dried sample was cut into small 15.8 mm plates and transferred to a glove box filled with argon gas to assemble CR2032 batteries. The rate and cycle of the assembled battery were studied with a battery tester (CT-4008 T) by galvanostatic discharge–charge cycling between 1 and 2.5 V, at a current rate of 0.2–10 C (1 C = 175 mAh g^−1^). Other electrochemical tests, including cyclic voltammetry (CV) at a scan rate of 0.5 mV s^−1^ and a scan range of 1.0–2.5 V, and electrochemical impedance spectroscopy (EIS), in a frequency range of 100 kHz to 10 mHz, were performed at the CHI760E electrochemistry workstation.

## Results and discussion

**Figure 2:**
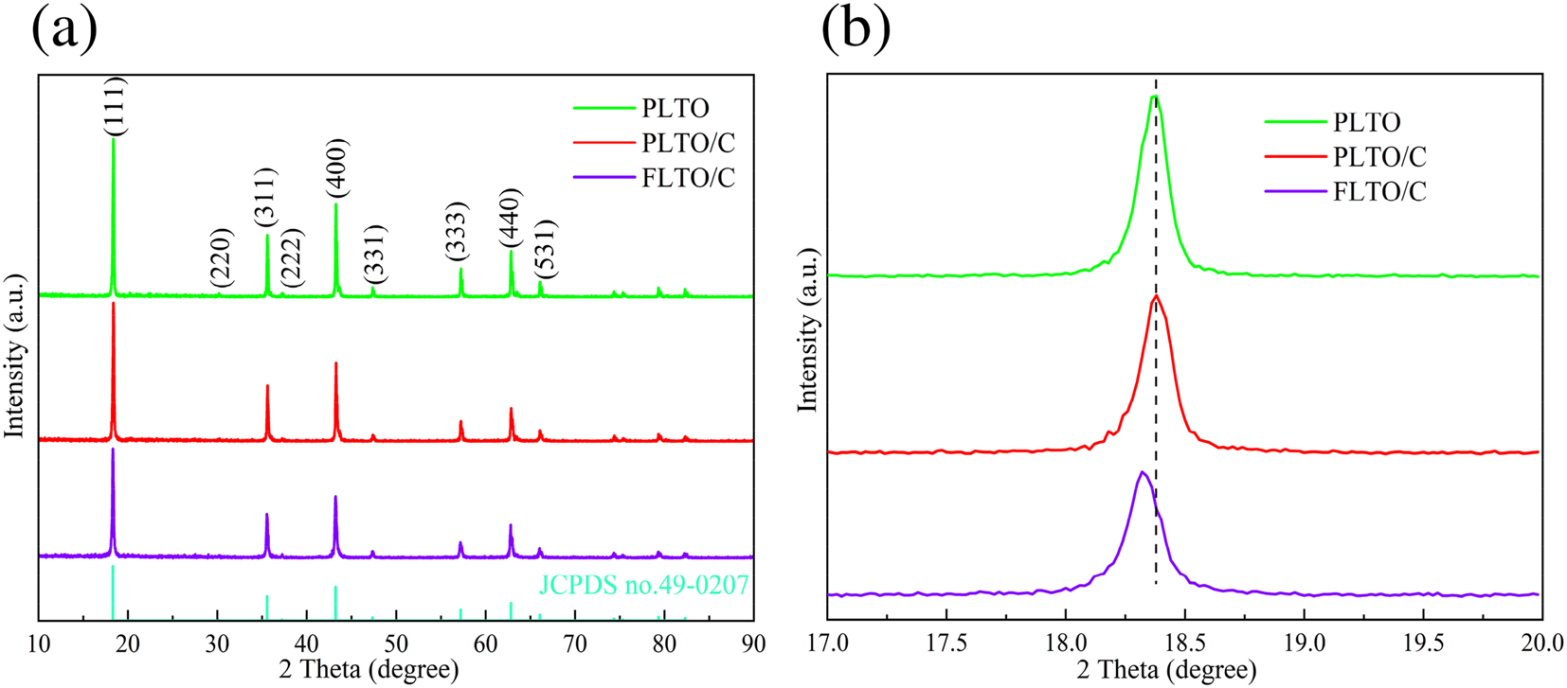
(**a**) XRD patterns and (**b**) magnified views of the (111) reflections of PLTO, PLTO/C, FLTO/C.

The crystal structure and phase composition of the FLTO/C, PLTO and PLTO/C were analyzed by XRD, and the results are shown in Fig. 2a. According to JCPDS 49-0207, the three LTO materials crystallized in the F__d3m space group. No peak of Fe-containing oxide is observed in the FLTO/C electrode material, demonstrating that Fe doping into the LTO lattice did not change the structure of LTO, or that the Fe content is low and undetected. Typically, when the ionic radius is close to the doped metal ion radius, the substitution reaction is more likely to occur or the dopant can enter the lattice gap to form an active center. The ionic radius of Fe^2+^ (0.78 Å)^24, 25^is similar to those of Li^+^ (0.76 Å)^26–28^ and Ti^4+^ (0.68 Å)^29–31^. In LTO structure, Li^+^ occupies the tetrahedral 8(a) position or octahedral 16(c) position; its occupying position can be reflected in the intensities of the (311) and (400) peaks. The relative strength ratio of the (311) and (400) peaks increases with the increase in Fe content, thus, reflecting the change in the Li^+^ ion position on Fe doping^32, 33^. The I_(311)_/I_(400)_ strength ratio of the FLTO/C electrode material is not much different from that of the original PLTO, indicating that Fe^2+^ is not doped into the Li site. Figure 2b shows a magnification of the XRD patterns of the three electrode materials for the (111) crystal plane. The peak of FLTO/C shifts to a smaller angle, indicating that the Fe^2+^ dopant entered the Ti site, further indicating that the carbon-coated Fe-doped LTO material had been successfully prepared.

**Figure 3:**
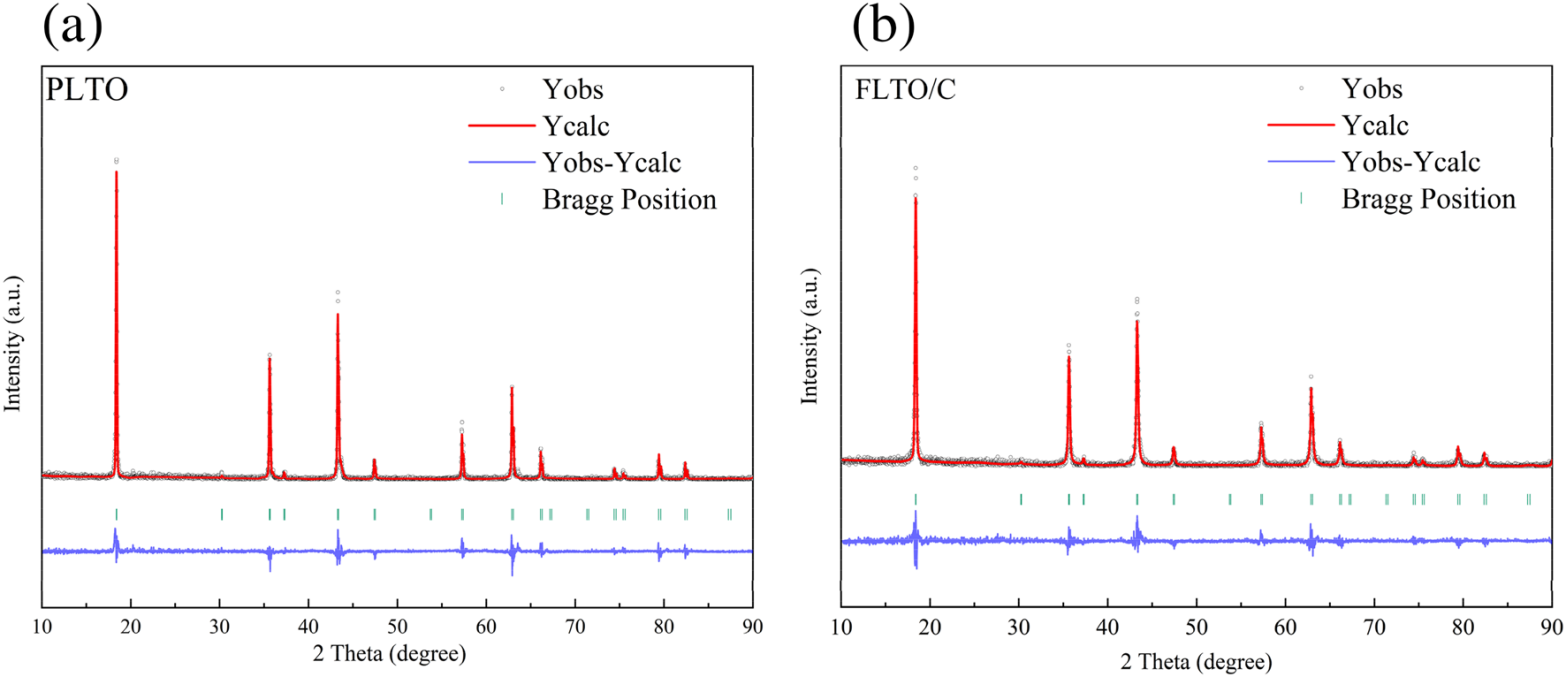
Experimental (black), calculated (red) and error profiles (blue) obtained from the Rietveld refinement of (**a**) PLTO and (**b**) FLTO/C.

The influence of Fe doping on the LTO structure was further examined by implementing Rietveld refinement on the Fullprof software, to calculate the crystal size, as depicted in Fig. 3a,b. The lattice parameters and other relevant information are summarized in Table 1. Results indicate that Fe substitution increased the lattice parameters from 8.35458 to 8.35504 Å. This shift can be attributed to the larger ionic radius of Fe^2+^ compared to Ti^4+^. The partial substitution of Ti^4+^ by Fe^2+^ increased the unit cell volume in the LTO structure. The accurate Fe concentration in the FLTO/C electrode material was obtained by ICP-OES analysis. The concentration of Fe in FLTO/C was approximately 0.27%, confirming the formation of Li_4_Ti_4.9865_Fe_0.135_O_12_/C.

**Table 1:** Cell parameters from the Rietveld refinement of PLTO and FLTO/C.

Sample	Lattice parameter (Å)	V (Å^3^)
PLTO	8.35458	583.141
FLTO/C	8.35504	583.238

**Figure 4:**
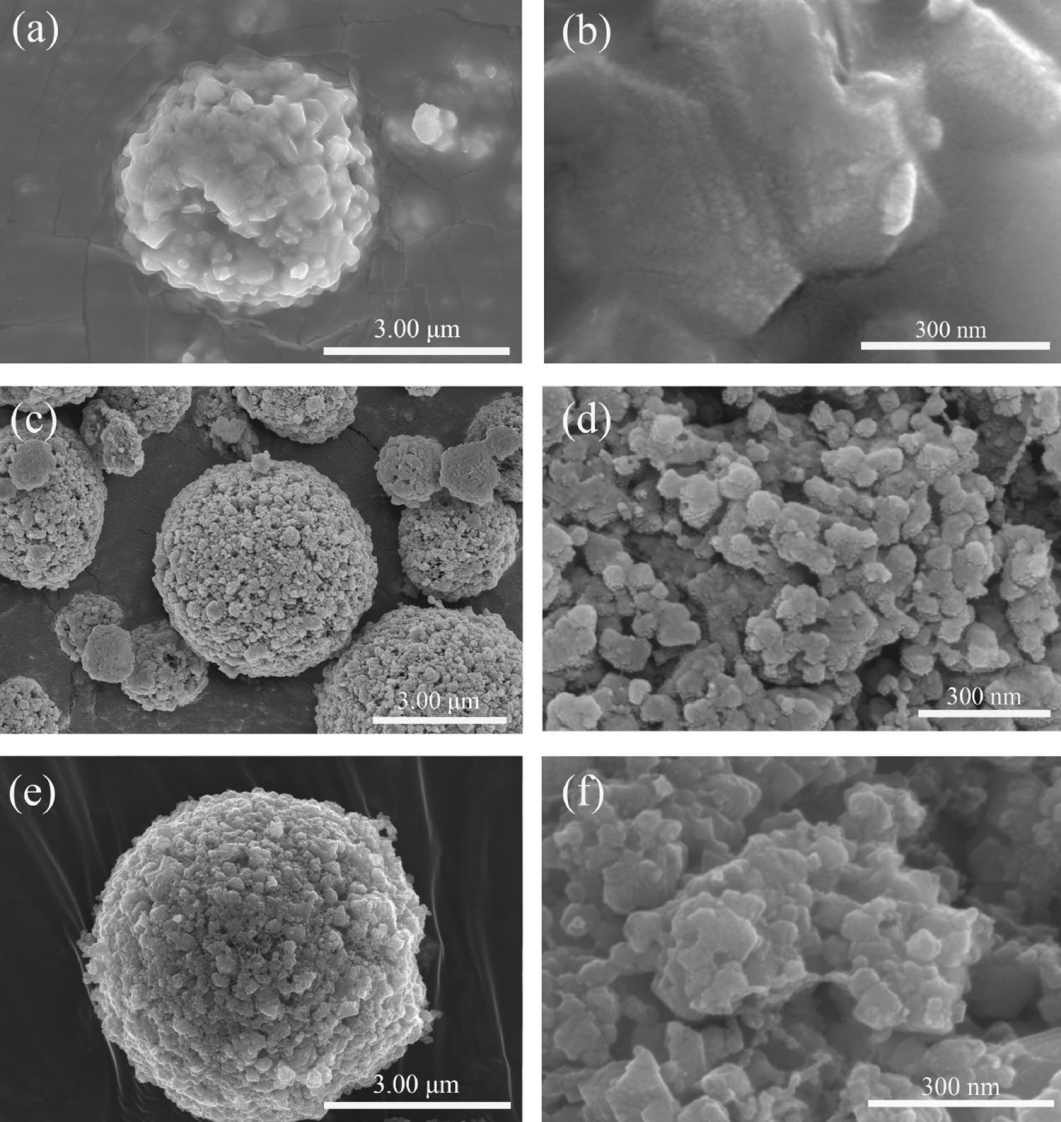
SEM images of (**a**, **b**) PLTO, (**c**, **d**) PLTO/C, and (**e**, **f**) FLTO/C.

The SEM images of PLTO, PLTO/C and FLTO/C in Fig. 4 show LTO microspheres of about 4–5 μm diameter. While the surface of the PLTO electrode material is smooth, the addition of 15% glucose roughens the surface and forms small primary particles and pores on the surfaces of PLTO/C and FLTO/C. This can be attributed to the addition of glucose, resulting in the thermal decomposition of glucose during calcination.

**Figure 5:**
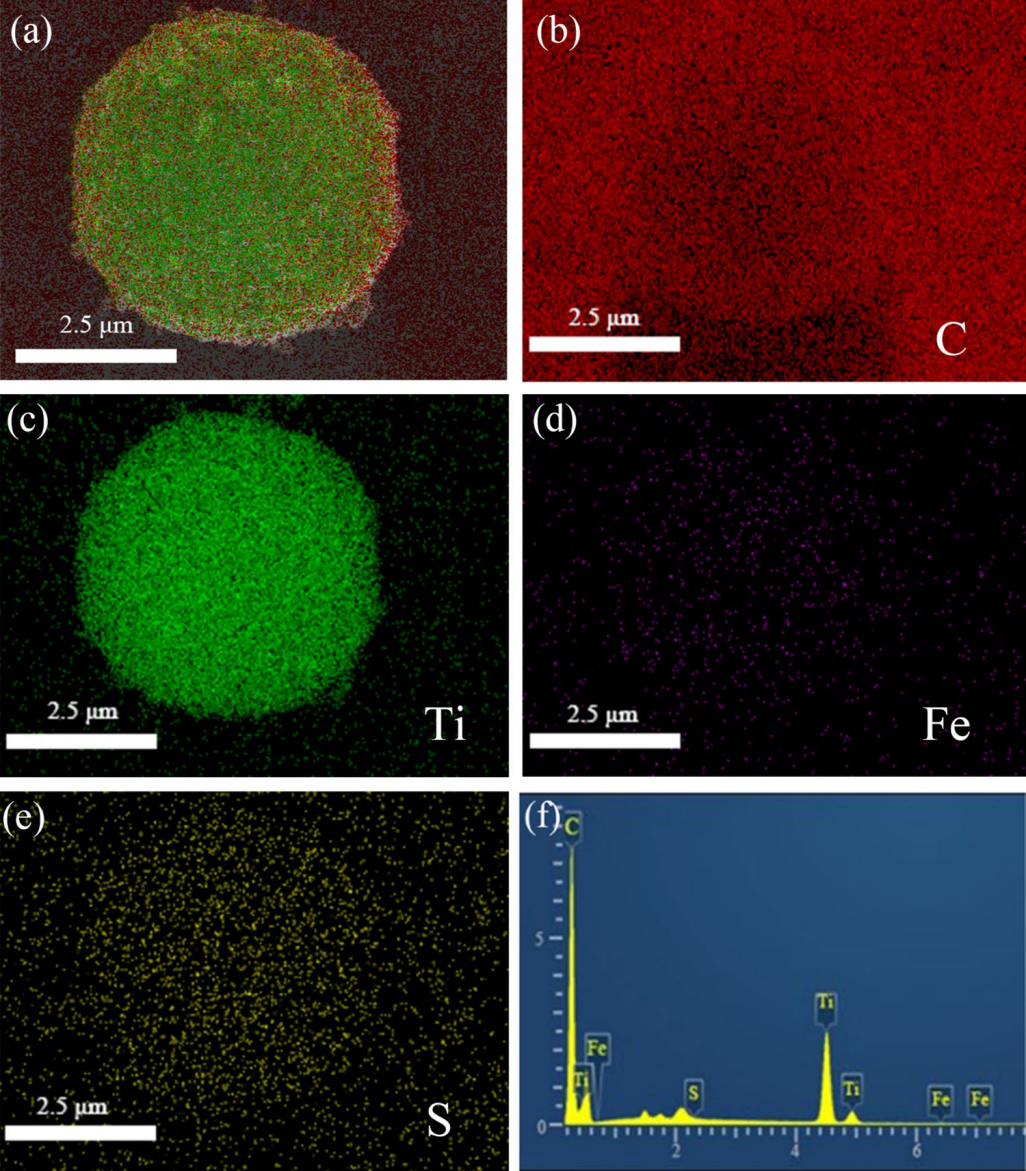
EDS elemental mappings of FLTO/C.

The EDS elemental mapping images of FLTO/C indicate that Ti, C, S and Fe were uniformly distributed in the electrode, as shown in Fig. 5. Trace amounts of surface sulfur on FLTO/C can be attributed to the residual sulfur from industrial H_2_TiO_3_, which is difficult to remove during the washing process. Figure 5d shows that the distribution of Fe is consistent with the distribution of Ti, O and C, indicating that Fe is uniformly distributed in the LTO crystal structure. The Fe ions are adsorbed by H_2_TiO_3_ with a strong binding force and good stability, which lays a foundation for subsequent doping reactions and uniform distribution. Meanwhile, XRD diffraction does not find the peak of iron oxide, so on the basis of the above discussion, we synthesized Fe-doped LTO/C microspheres.

**Figure 6:**
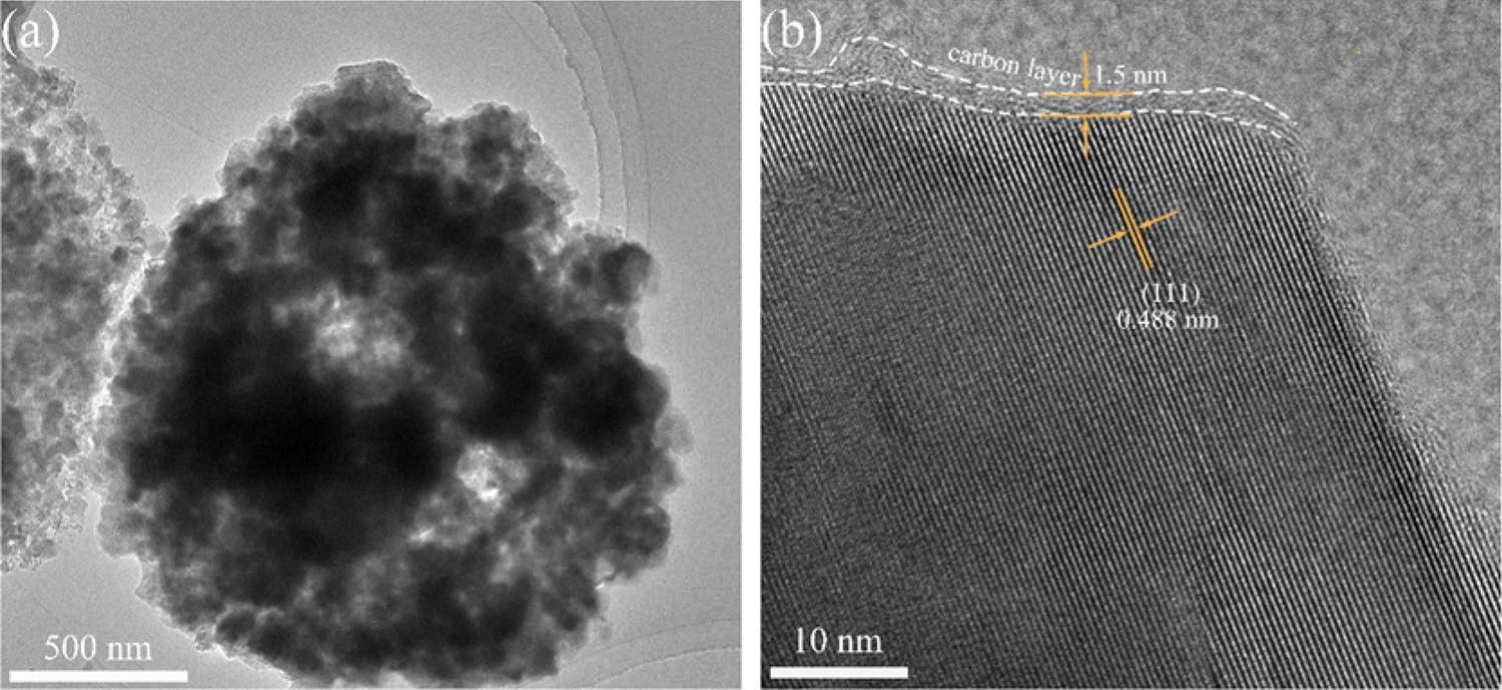
HRTEM images of FLTO/C.

The thickness of the carbon layer and lattice distance of FLTO/C were assessed using HRTEM (Fig. 6). The FLTO/C electrode material shows a reasonably uniform carbon layer with a thickness of approximately 1.5 nm. The (111) crystal plane of FLTO/C corresponds to a crystal plane spacing of 0.488 nm, while that of pure phase LTO is about 0.483 nm^34^. These results confirm that introducing Fe^2+^ into the LTO lattice increases the LTO lattice constant, aligning with the XRD results. An expanded crystal face spacing promotes the diffusion of Li^+^ culminating in better electrochemical performance of LTO.

**Figure 7:**
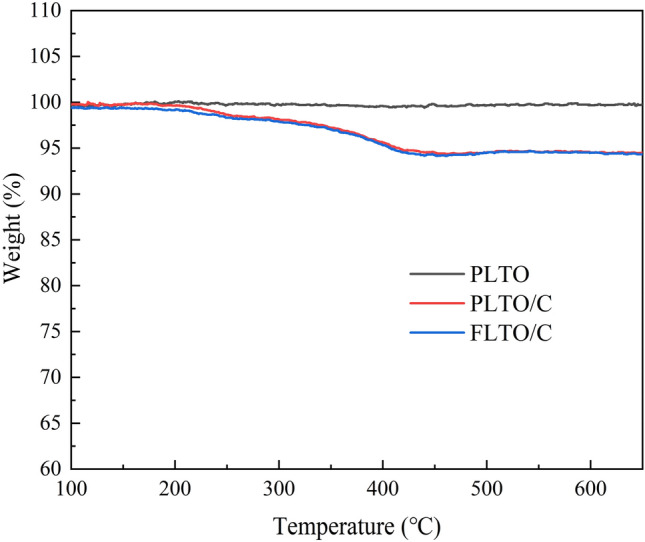
TG curves of PLTO, PLTO/C and LTO/C.

TGA analysis was used to show the relationship of weight loss with the temperature increase, as shown in Fig. 7. The thermal decomposition temperature of carbon is about 270–400 °C, and the mass loss indicates the coating ratio of carbon in the composite. In addition, TGA test of PLTO was also performed to ensure the accuracy of the conclusions. Therefore, based on the results of PLTO, PLTO/C and FLTO/C, it can be calculated that the carbon content in FLTO/C and PLTO/C is about 2.81 wt% and 2.77 wt%.

**Figure 8:**
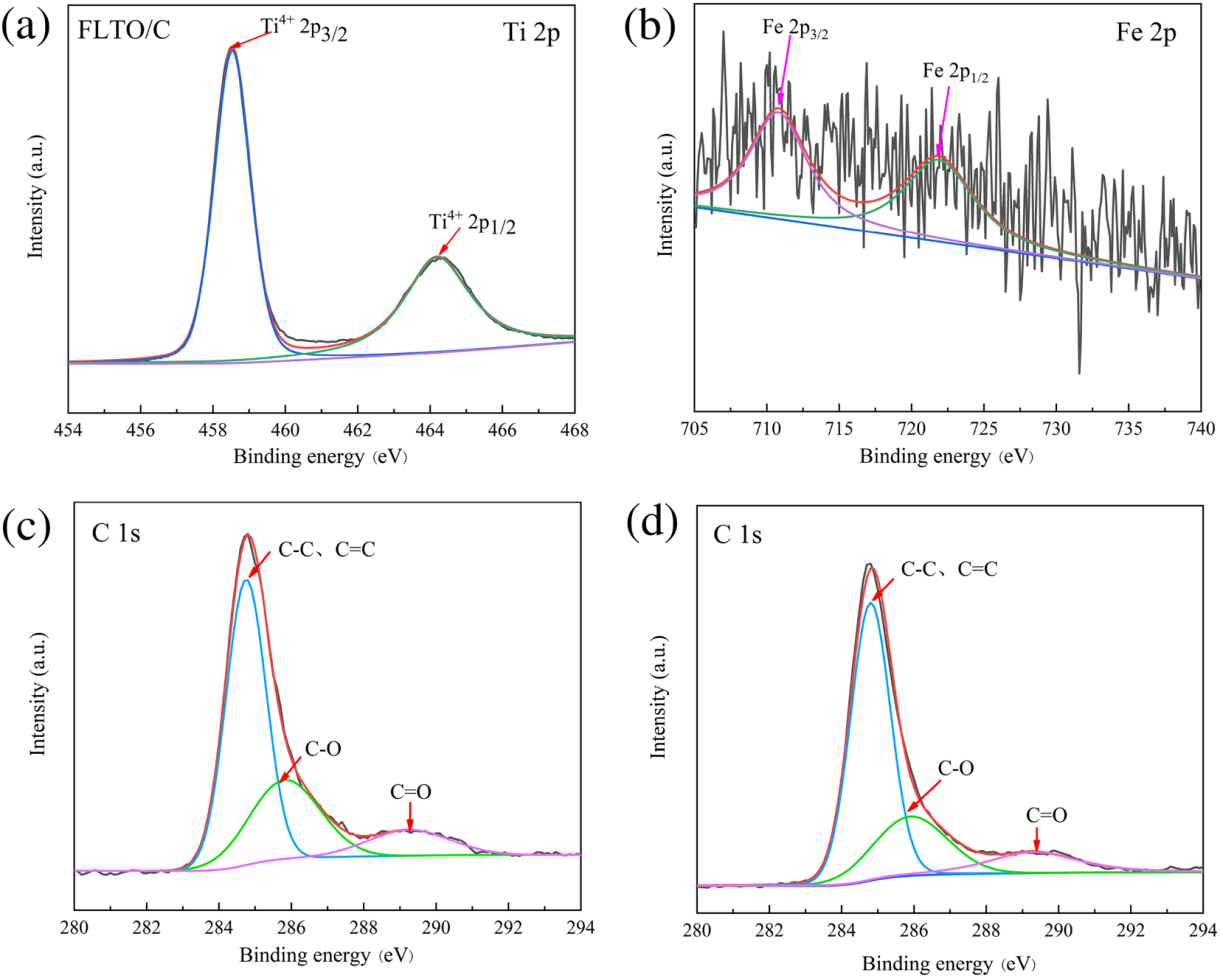
(**a**) Ti 2p XPS spectra of FLTO /C, (**b**) Fe 2p XPS spectra of FLTO/C. XPS C 1 s spectra of (**c**) FLTO/C, and (**d**) PLTO/C.

XPS was used to analyze the chemical state of Ti, C and Fe in FLTO/C, as shown in Fig. 8. The Ti 2p peaks can be assigned to Ti 2p_1/2_ (464.25 eV) and Ti 2p_3/2_ (458.5 eV)^35, 36^, corresponding to Ti^4+^ (Fig. 8a). Figure 8b shows the Fe 2p_1/2_ and Fe 2p_3/2_ peaks at 722.44 eV and 710.51 eV, respectively, indicating the presence of Fe^2+^^37, 38^. Figure 8c,d reveals the results of FLTO/C and PLTO/C after C1s partial peak fitting. The entire spectrum can be divided into three peaks, i.e., C–C and C=C near 284.8 eV, C–O at 285.9 eV, and C=O at 289.3 eV^39–41^. It is suggested that, when Fe is doped into the LTO lattice, the valence state and composition of C do not change.

**Figure 9:**
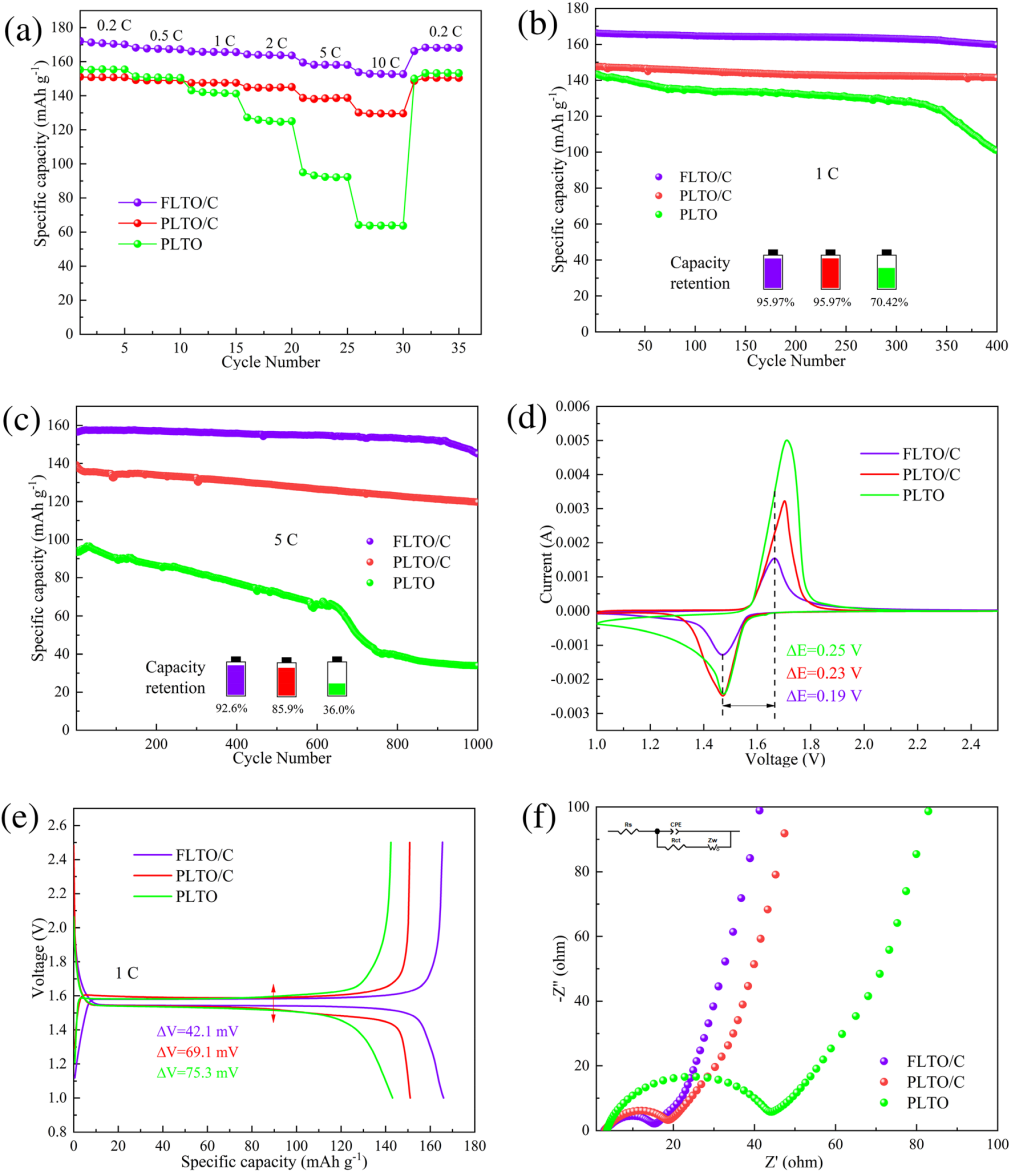
(**a**) Rate performance of PLTO, PLTO/C and FLTO/C, cycling curves at (**b**) 1 C, and (**c**) cycle curves at 5 C, (**d**) CV curves at 0.5 mV s^-1^, (**e**) galvanostatic discharging/charging curves at 1 C, (**f**) EIS spectra and the equivalent circuit model.

Half-cells were assembled using dual-modified FLTO/C composites as the active material and PLTO and PLTO/C as the control samples. Figure 9a shows the rate performance of PLTO, PLTO/C and FLTO/C, at current densities ranging from 1 to 10 C. At 0.2 C, 0.5 C, 1 C, 2 C, 5 C and 10 C, FLTO/C showed discharge capacities of 172.15, 168.21, 166.02, 164.27, 159.50, and 153.79 mAh g^−1^, respectively, significantly exceeding those of PLTO and PLTO/C as summarized in Table S1. These results show that FLTO/C has a higher specific capacity than 150 mAh g^−1^ over 2–10 C, significantly surpassing the limitations of higher rate performance of PLTO and PLTO/C.

The cycling performance of PLTO, PLTO/C and FLTO/C were tested at a current density of 1 C, as shown in Fig. 9b. After 400 cycles, the capacity retention rates of PLTO, PLTO/C and FLTO/C were 70.42%, 95.97% and 95.97%, respectively. Figure 9c illustrates the charge and discharge cycles at 5 C for PLTO, PLTO/C, and FLTO/C after 1000 cycles. The long-term cycling stability of PLTO and PLTO/C shows initial discharge capacities of 94.29 and 139.27 mAh g^−1^, respectively. After 1000 cycles, the capacity retention rates of PLTO and PLTO/C decrease to 35.99% and 85.91%, respectively, while their discharge capacities drop to 33.94 and 119.65 mAh g^−1^, respectively. After 1000 cycles at 5 C, FLTO/C exhibits an initial and residual specific capacity of 156.62 and 145.02 mAh g^−1^, respectively, with a corresponding capacity retention rate of 92.56% and an ultra-low capacity decay rate of 0.007% per cycle.

The above results show that due to the dual modification by Fe doping and carbon coating, the specific capacity and stability of LTO show dramatic improvement. This behavior can be explained as follows; when a certain amount of carbon is added, the surface of the prepared FLTO/C microspheres becomes coarser, leading to a larger specific surface area, and the surface holes become more obvious. The increase of these holes can further facilitate Li^+^ to pass through quickly. Moreover, after Fe doping, the cell parameters and cell volume of FLTO/C increase, which is conducive to the diffusion of Li^+^ and further improves the electronic conductivity^34^. Therefore, the combined modifications result in excellent rate performance and cycling stability, particularly in FLTO/C.

FLTO/C also demonstrates superior rate and cycling performance compared to control samples PLTO and PLTO/C, and other LTO composite materials and graphite materials, as shown in Table 2.

**Table 2:** Electrochemical performance comparison of FLTO/C with other LTO composites materials and graphite materials.

Materials	Current Rate (C)	Rate capacity (mAh g^-1^)	Current rate for cycling (C)	Cycle number	Capacity retention (%)	References
MFLTO	10	76.0	5	150	99.6	^42^
Li_4_Ti_4.95_Te_0.05_O_12_	10	120.3	5	100	98.2	^43^
B_0.3_-C@Li_4_Ti_5_O_12_	1	165.0	1	200	90.0	^44^
LTO-Ce	10	61.5	2	400	81.7	^45^
LTO-Ti	10	131.0	2	200	97.8	^46^
SGG	10	390.0	0.8	600	92.0	^47^
GGCC	10	478.8	0.5	325	86.9	^48^
FLTO/C	10	153.8	5	1000	92.6	Our work

CV was used to further study the kinetic behavior of the electrodes during the lithiation/de-lithiation process. Figure 9d shows the CV curves of PLTO, PLTO/C and FLTO/C at a sweep rate of 0.5 mV s^−1^ after 100 cycles, at 1 C. The proximity of the redox peak of FLTO, to that of PLTO, and the lack of heterodox peaks indicates that Fe doping does not alter the electrochemical reaction of the LTO substrate material. The peak separation values of PLTO, PLTO/C and electrode were 0.24 V and 0.23 V respectively, while the peak separation value of FLTO/C anode was the narrowest, only 0.19 V, so FLTO/C showed the smallest polarizability. Figure 9e displays the charge–discharge plateaus of the three electrode materials at 1 C. Compared with PLTO and PLTO/C, FLTO/C showed a slightly higher discharge voltage, the largest specific capacity and the smallest charge–discharge voltage difference (42.1 mV), which is consistent with the CV results.

The electrochemical behavior of the electrodes was studied through EIS and fitted to equivalent circuit models using the Z-view software, as shown in Fig. 9f. *R*_*s*_ represents the total resistance of the electrolyte, separator, and fluid collector, *R*_*ct*_ represents the charge transfer resistance, CPE represents a constant phase element, and *Z*_*w*_ represents the Warburg impedance, which correlates to the Li^+^ diffusion process. Figure S1 shows the impedance velocity (Z′ diagonal) of ω^−1/2^ for PLTO, PLTO/C, and FLTO/C. The fitting results (Table S2) and calculations for the Li^+^ diffusion coefficient (Equations S1 and S2) indicate that the total resistance (*R*_*s*_) values for PLTO, PLTO/C, and FLTO/C are 3.765, 3.323, and 3.175 Ω, respectively. In addition, the charge transfer resistance (*R*_*ct*_) for PLTO, PLTO/C, and FLTO/C are 35.34, 12.72, and 10.90 Ω, respectively. The calculated Li^+^ diffusion coefficients for PLTO, PLTO/C, and FLTO/C are 6.41 × 10^−15^, 9.45 × 10^−14^ and 5.95 × 10^−13^ cm^2^ s^−1^, respectively. Compared with the PLTO and PLTO/C electrode materials, the Li^+^ diffusion coefficient of FLTO/C increased by 1–2 orders of magnitude. Thus, the dual modification by Fe doping and carbon coating substantially enhanced the FLTO/C Li^+^ diffusion coefficients.

A four-point probe resistivity tester showed that the electronic conductivities of PLTO/C and FLTO/C were 2.15 × 10^−4^ and 8.50 × 10^−3^ S cm^−1^, respectively. Compared with the intrinsic conductivity of LTO, the electronic conductivities of the two electrode materials had significantly improved. These results demonstrate that the dual-modified LTO shows a considerably enhanced electrical conductivity. This improvement can be attributed mainly to the carbon coating on the surface of LTO. Moreover, the addition of Fe results in an increase in the unit cell size, which promotes the migration of electrons.

## Conclusions

In this study, a carbon-coated Fe-doped LTO composite material was successfully synthesized, under a carbon reduction atmosphere. Fe-containing industrial H_2_TiO_3_ was used as the titanium source to take advantage of the uniform distribution, strong binding force and high stability of the Fe ions, which were adsorbed by H_2_TiO_3_. The surface of the prepared microspheres of the carbon-coated Fe-doped LTO composite became coarser and had a larger specific surface area. This enabled the electrode material to have full contact with the electrolyte, thus, facilitating quick lithiation/de-lithiation of the Li^+^. Furthermore, Fe doping led to an increase in the cell parameters and cell volume of FLTO/C, facilitating the diffusion of Li^+^ and improving the electronic conductivity. The obtained FLTPO/C material exhibited excellent electrochemical performance, with a capacity retention rate of 95.97% even after 400 cycles, at a current density of 1 C. Most importantly, with an initial specific discharge capacity of 156.62 mAh g^−1^ at 5 C, its capacity retention rate was as high as 92.56% even after 1000 cycles, with a degradation of only 0.007% per cycle. The* R*_*ct*_ value of FLTO/C was 10.9 Ω, indicating a low charge transfer resistance. Its Li^+^ diffusion coefficient was 4.60 × 10^−12^ cm^2^ s^−1^, which is 1–2 orders of magnitude higher than pure phase PLTO and PLTO/C. As a result, LTO exhibited superior electrochemical activity and a higher Li^+^ diffusion rate when dually modified through Fe doping and carbon coating. In conclusion, LTO composites showing outstanding electrochemical performance were synthesized using a simple process and inexpensive raw materials. These results have significant breakthrough implications for the large-scale application and future development of LTO-based electrode materials.

### Supplementary Information

Supplementary Information.

## Data Availability

All data generated or analysed during this study are included in this published article [and its supplementary information files.
