# Supplementary material for: Synthesis of dual-modified Fe-doped and carbon-coated Li4Ti5O12 anode based on industrial H2TiO3 for Li-ion batteries
Source: Sci Rep. 2023 Sep 13;13:15118. doi: 10.1038/s41598-023-41830-x (PMC10499835; doi:10.1038/s41598-023-41830-x)
Supplement: Supplementary file 1 — Supplementary Information. [file 41598_2023_41830_MOESM1_ESM.pdf]

Supplementary Information

Synthesis of dual-modified Fe-doped and carbon-coated $Li_4Ti_5O_{12}$ anode based on industrial H_2TiO_3 for Li-ion batteries

Xinyu Jiang[a,b], Guangqiang Ma[a*], Qinmei Zhu[a], Hongwei Ge[a], Qiyuan Chen[a], Beilei Yan[a], Lin Deng[a], Congxue Tian[a] and Chuanbao Wu[a]

[a] *College of Biological and Chemical Engineering, Panzhihua University, 617000, Panzhihua, China*

[b] *Jiangxi Provincial Key Laboratory of Functional Molecular Materials Chemistry, School of Chemistry and Chemical Engineering, Jiangxi University of Science and Technology, Ganzhou 341000, P.R. China*

[*]Corresponding author.

E-mail address: magq3218@sina.com (G.-Q. Ma)

Table S1. PLTO, PLTO/C, and FLTO/C capacity at different rate

Sample	Discharge capacity at different rates（mAh g^{-1}）					
	0.2 C	0.5 C	1 C	2 C	5 C	10 C
PLTO	155.33	151.34	143.12	127.23	95.00	64.18
PLTO/C	152.05	149.19	147.55	144.98	138.70	130.08
FLTO/C	172.15	168.21	166.02	164.27	159.50	153.79

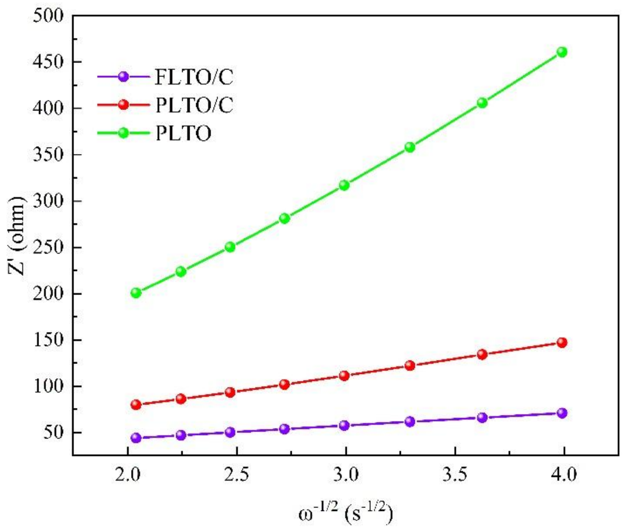

Fig.S1. Relationship between Z' and $\omega^{-1/2}$ of PLTO, PLTO/C, and FLTO/C samples.

The diffusion coefficient of Li^+ is calculated by the following equations[1,2]:

$$D_{Li+}=R^2T^2/(2A^2n^4F^4\sigma^2C^2) \quad \text{(Eq. S1)}$$

$$Zre = Rs + Rct + \sigma\omega^{-1/2} \quad \text{(Eq. S2)}$$

Table S2. *Rs* , R*ct* and σ values of PLTO, PLTO/C, and FLTO/C samples

Sample	*Rs* (Ω)	*Rct* (Ω)	σ (Ω cm^2 $s^{-0.5}$)
PLTO	3.765	35.34	133.287
PLTO/C	3.323	12.72	34.698
FLTO/C	3.175	10.9	13.827

Data availability

The data that support the findings of this study are available in the supplementary material of this article.

[1] B.-N. Yun, H.L. Du, J.-Y. Hwang. H.-G. Jung, Y.-K. Sun. Improved Electrochemical Performance of Boron-doped Carbon-coated Lithium Titanate as an Anode Material for Sodium-ion Batteries. J. Mater. Chem. A. 5 (2017) 2802–2810.

[2] X. Bai, *et al.* Towards Enhanced Sodium Storage of Anatase TiO_2 via a Dual-modification Approach of Mo Doping Combined with AlF_3 Coating. Nanoscale. 12 (2020) 15896–15904.
